# NRF2 deficiency leads to inadequate beta cell adaptation during pregnancy and gestational diabetes

**DOI:** 10.1016/j.redox.2025.103566

**Published:** 2025-02-24

**Authors:** Fatema Haidery, Luca Lambertini, Isabelle Tse, Sriya Dodda, Adolfo Garcia-Ocaña, Donald K. Scott, Sharon Baumel-Alterzon

**Affiliations:** a Diabetes, Obesity and Metabolism Institute, Icahn School of Medicine at Mount Sinai, New York, NY, USA; b Mindich Child Health and Development Institute, Icahn School of Medicine at Mount Sinai, New York, NY, USA; c Department of Molecular and Cellular Endocrinology, Arthur Riggs Diabetes & Metabolism Research Institute at City of Hope, Duarte, CA, USA

## Abstract

The late stages of mammalian pregnancy are accompanied by a mild increase in insulin resistance likely due to enhanced glucose demand of the growing fetus. Therefore, as an adaptive process to maintain euglycemia during pregnancy, maternal β-cell mass expands leading to increased insulin release. Defects in functional β-cell adaptive expansion during pregnancy can lead to gestational diabetes mellitus (GDM). While the exact mechanisms that promote GDM are poorly understood, GDM is associated with inadequate functional β-cell mass expansion and with a systematic increase of oxidative stress. Here, we show that NRF2 levels are upregulated in mouse β-cells at gestational day 15 (GD15). Inducible β-cell-specific *Nrf2* deleted (βNrf2KO) mice display reduced β-cell proliferation, increased β-cell oxidative stress and lipid peroxidation, compromised β-cell function, and elevated β-cell death, leading to impaired β-cell mass expansion and dysregulated glucose homeostasis towards the end of pregnancy. Importantly, the gestational hormone 17-β-estradiol (E2) increases NRF2 levels, and downregulation of NRF2 suppresses E2-induced protection of β-cells against oxidative stress, suggesting that E2 exerts its antioxidant effects through activation of NRF2 signaling in β-cells. Collectively, these data highlight the critical role of NRF2 in regulating oxidative stress during the adaptive response of β-cells in pregnancy and identify NRF2 as a potential therapeutic target for GDM treatment.

## Graphical abstract

**Figure:**
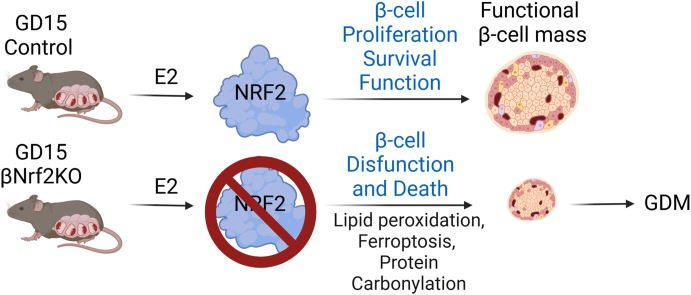

## 1 Introduction

The late stages of pregnancy are accompanied by a mild increase in maternal insulin resistance, likely due to enhanced glucose demand of the growing fetus [1,2]. Therefore, as an adaptive process to maintain euglycemia during pregnancy, maternal functional β-cell mass expands, leading to increased insulin release. Several cellular processes contribute to this expansion, including β-cell proliferation and β-cell neogenesis [1,2]. These processes are orchestrated by a specific combination of gestational hormones, growth factors and neurotransmitters, including lactogens (prolactin, placental lactogens), estrogen, progesterone, hepatocyte growth factor (HGF), epidermal growth factor receptor (EGFR), and serotonin. Once bound to their receptors, these factors trigger multiple mitogenic signaling pathways resulting in the expansion of β-cell mass [[1], [2], [3]].

Defects in the adaptive expansion of functional β-cells during pregnancy can lead to the development of gestational diabetes mellitus (GDM) [1]. In the U.S., the prevalence of GDM is approximately 7.6 % of all pregnancies [4], with a sharp increase of 10–100 % across various racial and ethnic groups over recent decades [5,6]. GDM increases the risk of developing Type 2 diabetes (T2D) post-pregnancy in the mother and perturbs fetal development [4,7]. While the exact mechanisms that promote GDM are poorly understood, GDM is associated with inadequate functional β-cell mass expansion [1,8,9] and with a systematic increase of oxidative stress [[10], [11], [12], [13], [14], [15]]. Yet studies analyzing the effect of oxidative stress on the adaptive expansion of β-cells during pregnancy are lacking.

We have previously shown that the master regulator of oxidative stress, nuclear factor erythroid 2-related BZIP transcription factor 2 (*Nfe2l2*), more commonly known as NRF2, is necessary for β-cell adaptive expansion in situations of increased insulin demand, such as diet-induced obesity (DIO) and neonatal growth [16,17]. In addition, several single nucleotide polymorphisms (SNPs) in *NRF2*, are associated with T2D in the human population [[18], [19], [20], [21], [22]]. A recent multiomics study, combining proteomics and transcriptomics analysis of mouse islets predicted the upregulation of NRF2 signaling in islets of pregnant rodents [23]. We thus hypothesized that NRF2 might be required for the adaptive response of β-cells during pregnancy and that alterations of the NRF2 signaling may result in the development of GDM.

Here, we show that NRF2 levels are upregulated in mouse β-cells at gestational day 15 (GD15). Importantly, mice with β-cell-specific *Nrf2* deletion (βNrf2KO) displayed a remarkable increase in β-cell oxidative stress, elevated β-cell death, and blunted β-cell proliferation. These effects resulted in attenuated pregnancy-induced β-cell mass expansion, reduced plasma insulin, impaired glucose tolerance, and compromised β-cell function, all of which are characteristics of GDM. RNA sequencing (RNAseq) analysis of islets from βNrf2KO mice at GD15 revealed reduced expression of genes associated with cell cycle, mitochondrial activity, and enzymes that inhibit lipid peroxidation, ferroptosis and protein carbonylation in β-cells. Importantly, we found that the gestational hormone 17-β-estradiol (E2) increases NRF2 levels, and downregulation of NRF2 suppresses E2-induced protection of β-cells against oxidative stress, suggesting that E2 exerts its antioxidant effects in β-cells through activation of the NRF2 pathway. Taken together, these data highlight NRF2 as an essential regulator of adaptive functional β-cell expansion during pregnancy and a potential therapeutic target for treating GDM.

## 2 Results

### 2.1 NRF2 expression is upregulated in mouse β-cells at GD15

**Fig. 1:**
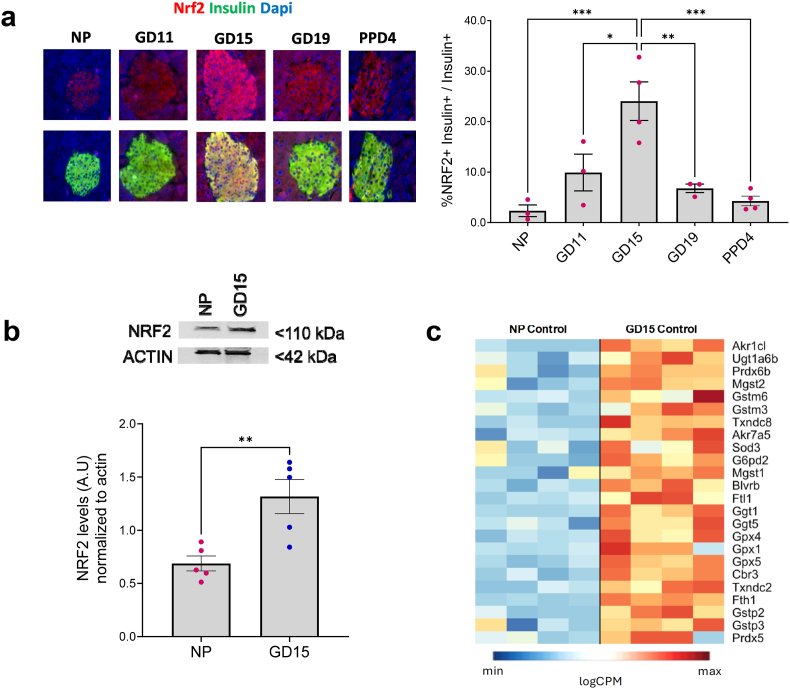
**NRF2 expression is upregulated in mouse β-cells at GD15**. (a) Pancreatic sections from C57BL6 female mice at different gestational days were embedded and immunolabeled for NRF2 and insulin (scale bar 50 μM). The percentage of nuclear NRF2- and insulin-positive cells were calculated. (b) Islets from NP or GD15C57BL6 female mice were subjected to immunoblotting using a NRF2 and actin antibodies. NRF2 levels were quantified using ImageJ software. (c) Expression heatmap of normalized log_2_ counts per million (logCPM) of a set of selected antioxidant genes from the RNAseq that was performed on islets isolated from control NP and GD15 mice. Data are the means ± SEM for n = 3–5, ∗*p* < 0.05, ∗∗*p* < 0.01, ∗∗∗*p* < 0.001.

A recent report predicted that the NRF2 signaling pathway might be upregulated in mouse islets during pregnancy [23]. We thus characterized NRF2 levels in islets during gestation and analyzed the role of NRF2 in β-cell adaptation during pregnancy. For this purpose, NRF2 and insulin immunolabelling was performed in pancreas sections of non-pregnant (NP) or pregnant C57BL6 mice at GD11, GD15, GD19 and 4 days postpartum (PPD4). As shown in Fig. 1a, NRF2 levels significantly increased (10-fold) in β-cells at GD15 compared to non-pregnant mice. During later stages of pregnancy, NRF2 levels dramatically decreased (72 % reduction in GD19 compared to GD15), returning to their basal levels postpartum (82 % reduction in PPD4 compared to GD15). Analysis of NRF2 levels in isolated islets by immunoblotting (Fig. 1b) confirmed the upregulation of NRF2 protein at GD15, without a corresponding increase in mRNA levels, as shown by RNAseq analysis of isolated islets from NP and GD15 mice (Supp. Fig. 1a), indicating post-transcriptional regulation of NRF2. No effect on the levels of the NRF2-inhibitor KEAP1, was observed in islets at GD15 (Supp. Fig. 1b). According to the RNAseq analysis, the expression of several NRF2 antioxidant target genes was upregulated in islets at GD15 compared to NP, including genes encoding various members of the antioxidant enzyme family *Prdx, Gstm, Gstp,* and *Gpx* (Fig. 1c; Supp. Table 1). Notably, not all NRF2 target genes were upregulated as some were either downregulated (E.g; *Nqo1, Hmox1,* and *Txnrd1*) or not significantly altered (E.g; *Sod1* and *Cbr1*) by pregnancy (Supp. Table 1). These results indicate that NRF2 protein (but not mRNA) levels and selected genes in NRF2 antioxidant signaling pathway are upregulated during pregnancy and therefore may play a role in the adaptation of the β-cell during pregnancy.

### 2.2 NRF2 is essential for adaptive expansion of β-cell mass during pregnancy

**Fig. 2:**
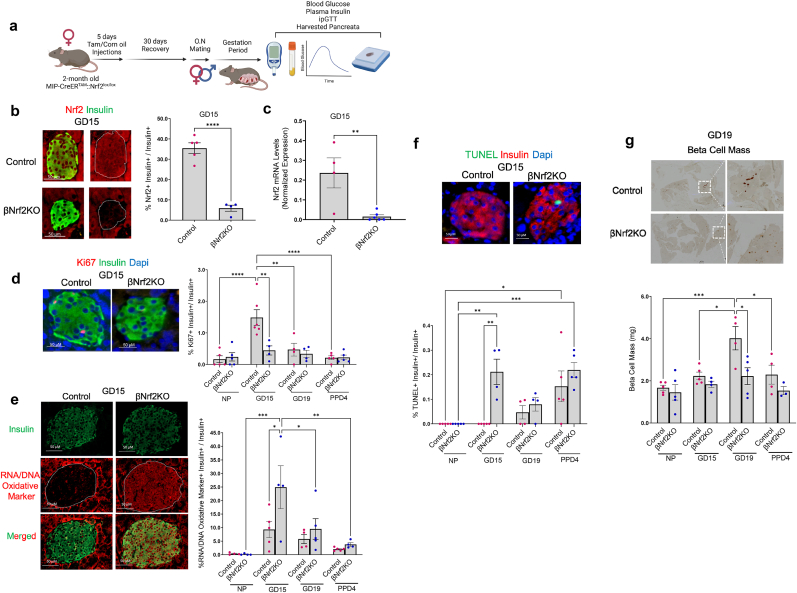
**NRF2 is essential for adaptive expansion of β-cell mass during pregnancy.** (a) Two-month-old female MIP-CreER^TAM^::Nrf2^lox/lox^ mice were daily intraperitoneally injected with tamoxifen (Tam to generate βNrf2KO) or corn oil (“control”) for 5 consecutive days, followed by a 30-day washout period before mating. At the end of the gestational period (GD15, GD19 or PPD4), blood glucose and plasma insulin were taken, glucose tolerance test (ipGTT) was performed and pancreata were harvested for further immunolabelings. This figure was created using BioRender.com. (b). Pancreatic sections from GD15 control or βNrf2KO female mice were immunolabeled for NRF2 and insulin (scale bar 50 μM). The percentage of nuclear NRF2- and insulin-positive cells were calculated. (c) Nrf2 mRNA levels in islets from GD15 control or βNrf2KO were quantified using qPCR and normalized to actin. (d) Pancreatic sections from control or βNrf2KO female mice at different gestational days were immunolabeled for Ki67 and insulin (scale bar 50 μM), or (e) oxidative stress marker (RNA/DNA oxidative marker) and insulin (scale bar 50 μM), or (f) TUNEL assay and insulin (scale bar 50 μM). Percentage of Ki67-positive or TUNEL-positive or oxidative stress marker-positive and insulin-positive cells were calculated. (g) Pancreatic sections from control or βNrf2KO females at different gestational days were immunolabeled for insulin. Data are means ± SEM for n = 3–10. ∗*p* < 0.05, ∗∗*p* < 0.01, ∗∗∗*p* < 0.001, ∗∗∗∗*p* < 0.0001.

As a key driving force of maternal β-cell expansion in rodents, β-cell proliferation gradually increases during early stages of pregnancy, peaking at GD15, and progressively declining to basal levels postpartum [[24], [25], [26]]. Since NRF2 follows a similar temporal pattern of expression, we addressed whether NRF2 plays a role in β-cell proliferation during pregnancy. For this purpose, we used our previously established tamoxifen-induced β-cell-specific *Nrf2* deletion mouse model (βNrf2KO) [16]. As tamoxifen can cause pregnancy complications [27], βNrf2KO female mice were treated with intraperitoneal injections of tamoxifen or corn oil as control [16] for 5 consecutive days, followed by a 30-day washout period [[28], [29], [30], [31]] before mating (Fig. 2a). As expected, the number of embryos per mouse did not differ between pregnant βNrf2KO and control mice (Supp. Fig. 1c), suggesting that the tamoxifen regime did not affect pregnancy outcomes. Blood glucose, plasma insulin and intraperitoneal glucose tolerance test (IPGTT) were then performed, and pancreata were harvested at the end of several gestational periods (GD15, GD19 or PPD4) for immunolabeling. NRF2 and insulin immunolabeling showed an 84 % decrease in β-cell NRF2 levels in islets of βNrf2KO mice compared to control mice at GD15 (Fig. 2b). *Nrf2* downregulation was also observed at the mRNA level in islets from GD15 βNrf2KO mice (Fig. 2c). Ki67 and insulin immunolabeling of control mouse pancreas showed the expected increase in β-cell proliferation at GD15 compared to NP mice (9-fold), followed by a decrease to basal levels at GD19 and PPD4 (Fig. 2d). However, the increase in β-cell proliferation at GD15 was blunted in βNrf2KO mice, indicating that NRF2 plays an important role in supporting β-cell proliferation during pregnancy.

We next tested whether deletion of *Nrf2* in β-cells would result in changes of redox balance. Accordingly, we performed immunolabeling of pancreatic sections from these mice with antibodies recognizing either insulin or the oxidized nucleic acid species 8-OHdG, 8-OHG and 8-oxo-Gua, common RNA/DNA oxidative stress markers in β-cells [16,17] (Fig. 2e). A mild, non-significant increase in oxidative stress was observed in β-cells of control mice at GD15 compared to control non-pregnant mice. However, oxidative stress levels were significantly elevated in β-cells of βNrf2KO mice at GD15 compared to control pregnant mice and were reduced over time. We then hypothesized that deletion of *Nrf2* in βNrf2KO mice would promote β-cell death due to increased oxidative stress. To test this hypothesis, we immunolabeled pancreata for insulin and performed TUNEL assay (Fig. 2f). In agreement with β-cells undergoing a surge of β-cell death postpartum [24,25,32], β-cells from both control and βNrf2KO mice displayed a similar increase in β-cell death at PPD4. Since oxidative stress is normalized postpartum, we speculate that PPD4 β-cell death is not a direct result of oxidative stress. However, as expected, βNrf2KO mice at GD15 displayed a substantial increase in β-cell death (100 % increase) compared to control pregnant mice which correlated with the observed increase in oxidative stress. These findings highlight NRF2's role in maintaining adequate redox balance in β-cells during pregnancy, by protecting them from cell death at GD15.

To test whether NRF2 plays a role in β-cell mass expansion during pregnancy, pancreata from pregnant control and βNrf2KO mice were immunolabeled for insulin and analyzed for β-cell mass by histomorphometry analysis (Fig. 2g). β-Cell mass reaches its maximal expansion in pregnant rodents at GD19 [24,25]. As shown in Fig. 2g, control mice displayed a significant expansion of β-cell mass at GD19 (2.4-fold compared to NP mice). Yet, this increase was not observed in βNrf2KO mice, suggesting that NRF2 is required for adaptive β-cell mass expansion during pregnancy.

### 2.3 NRF2 is essential for adaptive β-cell function and normal glucose homeostasis during pregnancy

**Fig. 3:**
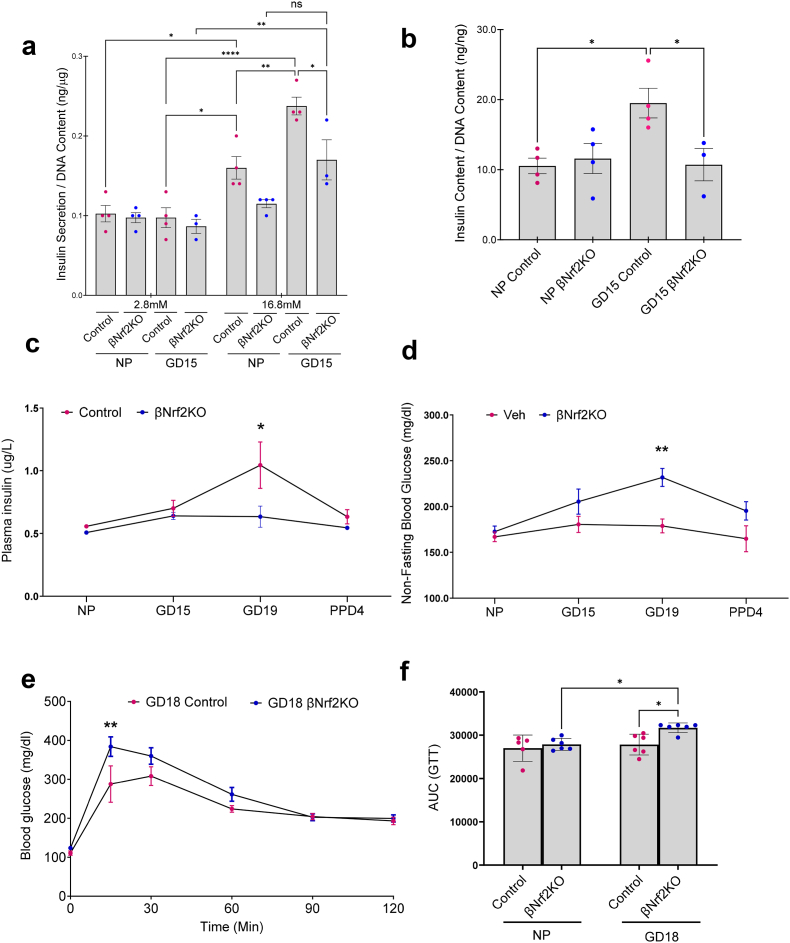
**NRF2 is essential for adaptive β-cell function and normal glucose homeostasis during pregnancy.** (a) Glucose-stimulated insulin secretion (GSIS) was performed on islets isolated from NP or GD15 control or βNrf2KO mice. (b) Insulin content was measured in these islets. (c) Plasma insulin and (d) non-fasting blood glucose were measured at different gestational days, and (e,f) IPGTT was performed at GD18. Data are means ± SEM for n = 3–10. ∗*p* < 0.05, ∗∗*p* < 0.01.

Maternal β-cell function is enhanced during pregnancy [[33], [34], [35]]. Not surprisingly, GDM is associated with impaired β-cell function [36,37]. To address whether NRF2 participates in the regulation of β-cell function during pregnancy, glucose-stimulated insulin secretion (GSIS) was performed in islets isolated from non-pregnant or GD15 control and βNrf2KO mice (Fig. 3a). Our data show that GSIS was enhanced at GD15 in both control and βNrf2KO mouse islets. However, while insulin secretion at low glucose concentration (2.8 mM) was not significantly different between GD15 control and GD15 βNrf2KO mouse islets, at high glucose concentration (16.8 mM), insulin secretion in GD15 βNrf2KO mouse islets was significantly lower than in GD15 control mouse islets, suggesting impaired insulin secretion response to high glucose levels in GD15 βNrf2KO mouse islets. Moreover, at 16.8 mM glucose, insulin secretion was significantly increased in GD15 control compared to NP control mouse islets, whereas no significant increase was observed in GD15 βNrf2KO compared with NP βNrf2KO mouse islets. In contrast to βNrf2KO mouse islets from male mice [16], NP female βNrf2KO mouse islets showed impaired insulin secretion response to high glucose levels. Additionally, islets from GD15 βNrf2KO mice exhibited significantly reduced insulin content (62 % reduction) compared to GD15 control mice (Fig. 3b). Collectively, these findings indicate that NRF2 is essential for β-cell function during pregnancy and basal conditions in females.

To determine the effect of β-cell-specific *Nrf2* deletion on glucose homeostasis, plasma insulin and blood glucose were analyzed at different gestational days, and a glucose tolerance test was performed at GD18. As expected, the increase in β-cell mass and insulin secretion in control pregnant mice correlated with an increase in plasma insulin levels (Fig. 3c), sustained normal blood glucose levels (Fig. 3d) and improved glucose tolerance (Fig. 3e and f). Conversely, βNrf2KO mice displayed reduced plasma insulin levels (Fig. 3c), hyperglycemia (Fig. 3d) and decreased glucose tolerance during pregnancy (Fig. 3e and f). These findings demonstrate that NRF2 is required for normal glucose homeostasis during pregnancy and dysregulation of NRF2 signaling in β-cells leads to the development of GDM.

### 2.4 NRF2 deficiency reduces the expression of enzymes that block lipid peroxidation, ferroptosis and protein carbonylation in β-cells of pregnant mice

**Fig. 4:**
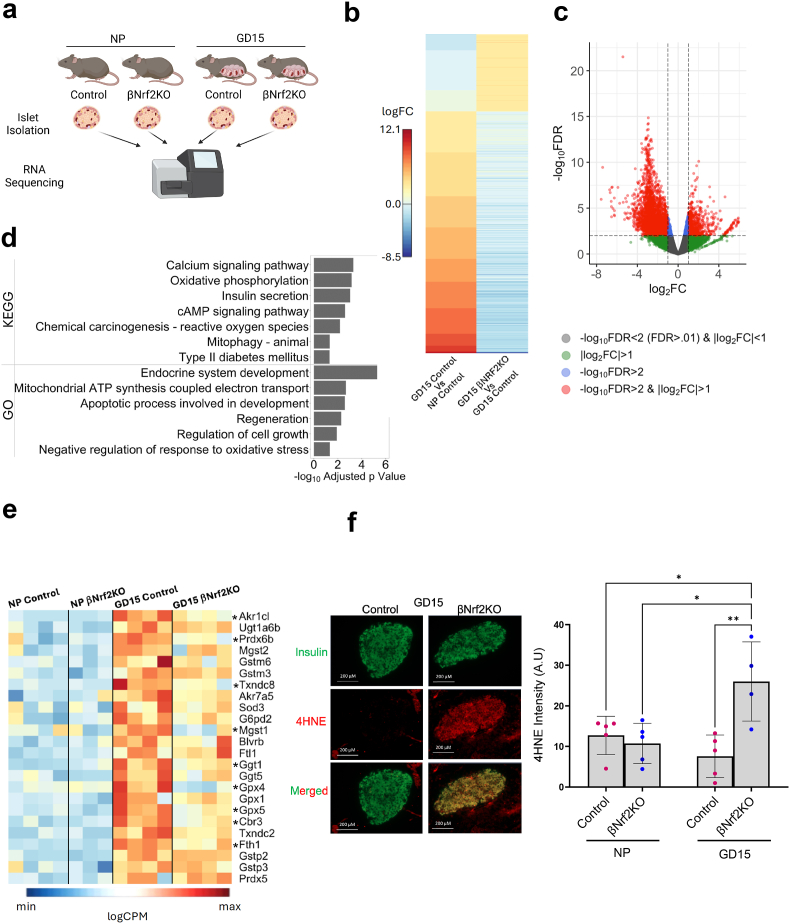
**NRF2 dysregulation reduces the expression of enzymes that block lipid peroxidation, ferroptosis and protein carbonylation in β-cells of pregnant mice.** (a) Islets from control or βNrf2KO NP or GD15 mice (3–4 samples per condition) were isolated and sent for RNAseq. This figure was created using BioRender.com. (b) Comparison of differentially expressed genes in GD15 controls versus NP controls mice (left column) and comparison of differentially expressed genes in GD15 βNrf2KO versus GD15 control mice (right column). (c) Volcano plot of the GD15 βNrf2KO versus GD15 Control differential gene expression. (d) Bar chart of the key pathways populated by the differentially expressed genes using GO biological process and KEGG. (e) Heatmap of the logCPMs for a subset of selected antioxidant genes across the 4 experimental conditions. Some of these data were used to generate Fig. 1c. Asterisks indicate significance between βNrf2KO GD15 and GD15 control. (f) Pancreatic sections from NP or GD15 control or βNrf2KO female mice were immunolabeled for 4HNE and insulin (scale bar 200 μM). 4HNE intensity was quantified using ImageJ software. Data are means ± SEM for n = 4–5. ∗*p* < 0.05, ∗∗*p* < 0.01.

To study the genes and pathways regulated by NRF2 in β-cells during pregnancy, RNAseq analysis of isolated islets from NP and GD15 in control and βNrf2KO mice was performed (Fig. 4a). Comparison of differentially expressed genes in GD15 controls versus NP controls mice (Fig. 4b left column) to differentially expressed genes in GD15 βNrf2KO versus GD15 control mice (Fig. 4b right column) shows an inverted trend, indicating that *Nrf2* deletion interferes with the expression of a subset of pregnancy-related genes. Overall, when comparing GD15 βNrf2KO vs GD15 controls, 435 genes were significantly upregulated, and 1044 were significantly downregulated (Fig. 4b right column and Fig. 4c). Notably, pathway analysis using Gene Ontology (GO) biological process, and the Kyoto Encyclopedia of Genes and Genomes (KEGG) highlighted multiple pathways affected by NRF2 deficiency during pregnancy (Fig. 4d). These include insulin secretion (and insulin secretion associated pathways such as calcium and cAMP signaling); regeneration and regulation of cell growth [supported by reduced expression of several cell cycle regulators (Supp. Fig. 1d)]; apoptotic process; mitochondrial function [including mitophagy, mitochondrial ATP synthesis and oxidative phosphorylation, supported by the reduced expression of several mitochondrial genes (Supp. Fig. 1e)]; T2D; and oxidative stress.

Oxidative stress leads to accumulation of reactive oxygen species (ROS) that can target the cell membrane, giving rise to oxidative chain reactions of w3 and w6 polyunsaturated fatty acids (lipid peroxidation) that are present in membrane phospholipids. The final products of this process are reactive aldehydes [38]. Once formed, these reactive molecules are extremely toxic to the cell, enabling formation of irreversible bonds with proteins, termed “protein carbonylation”, that impairs protein function and stability [[39], [40], [41]]. One of the most studied reactive aldehydes is 4-hydroxy-2-noneal (4HNE), which plays a significant role in many pathological conditions including diabetes [[42], [43], [44], [45]]. Interestingly, several genes that participate in preventing formation and protection against reactive aldehydes were downregulated in islets from GD15 βNrf2KO mice compared to islets from GD15 control mice (Fig. 4e). These include peroxiredoxin 6b (*Prdx6b*), thioredoxin domain-containing protein 8 (*Txnrdc8*), and other antioxidants that help neutralizing ROS thus preventing lipid peroxidation. In addition, glutathione peroxidase 4 (*Gpx4*) and ferritin heavy chain 1 (*Fth1*), encoding for proteins involved in inhibiting Ferroptosis-induced cell death driven by iron-dependent lipid peroxidation [46,47], were also downregulated (Fig. 4e). Lastly, three genes encoding for proteins that directly detoxify free reactive aldehydes, such as aldo-keto reductase family 1 member C-like (*Akr1cl*), microsomal glutathione transferase 1 (*Mgst1*) and carbonyl reductase 3 (*Cbr3*) [39,48,49], were also downregulated in islets from GD15 βNrf2KO mice. In support of these findings, 4HNE and insulin immunolabeling (Fig. 4f) showed increased 4HNE levels in β-cells of GD15 βNrf2KO mice compared to GD15 control mice. These results suggest that NRF2 deficiency enhances lipid peroxidation and consequently ferroptosis and protein carbonylation, limiting the expansion of functional β-cell mass during pregnancy.

### 2.5 17-β-estradiol (E2) exerts its antioxidant effect on β-cells through activation of the NRF2 signaling

**Fig. 5:**
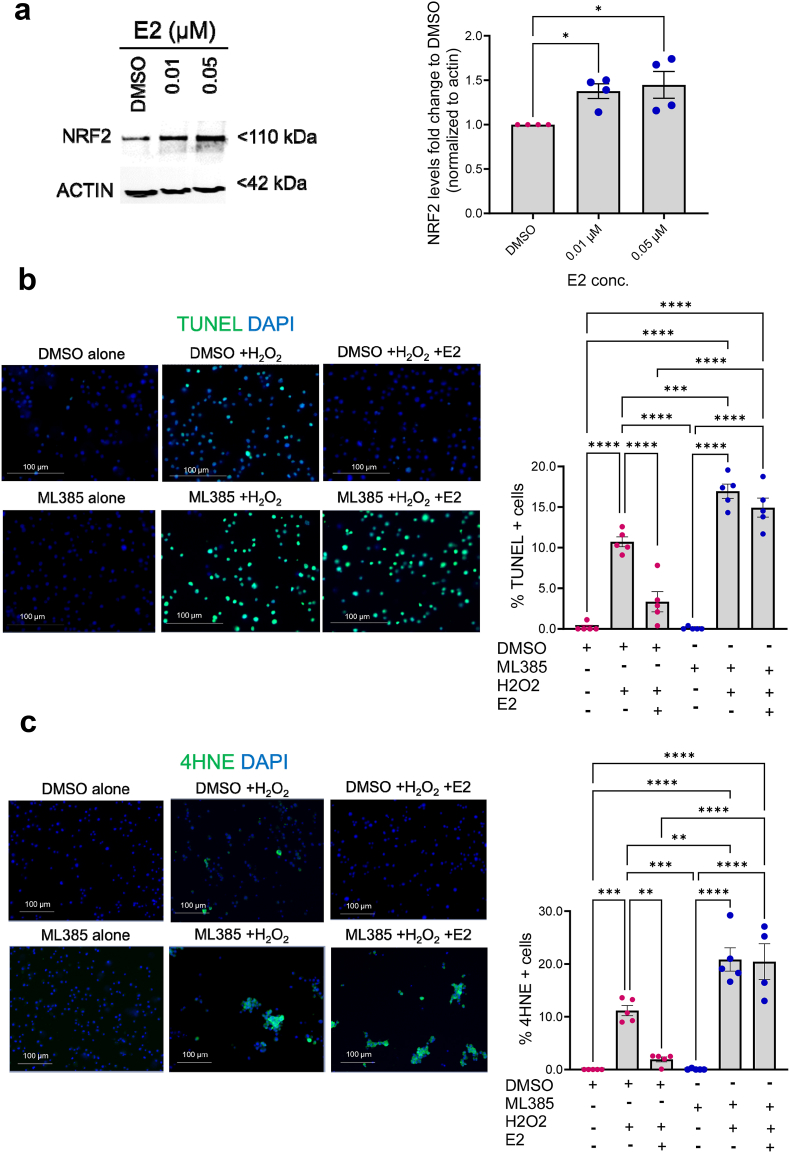
**The gestational hormone 17-β-estradiol (E2) exerts its antioxidant effect on β-cells through activation of the NRF2 signaling.** (a) Min6 β-cells were incubated with DMSO or E2 for 18 h and immunoblotting using NRF2 antibody was performed. NRF2 levels were quantified using ImageJ software. (b) Min6 β-cells were incubated with DMSO or 5 μM ML385, with or without 0.01 μM E2 for 72 h. Cell were then exposed to 100 μM H_2_O_2_ for 6 h. TUNEL assay or (c) immunolabeling for 4HNE and insulin were performed (scale bar 100 μM). Data are means ± SEM for n = 4–5. ∗*p* < 0.05, ∗∗*p* < 0.01, ∗∗∗*p* < 0.001, ∗∗∗∗*p* < 0.0001.

Since NRF2 levels are upregulated in β-cells during pregnancy, we next investigated whether gestational hormones affect NRF2 levels. For this purpose, Min6 β-cells were incubated with physiological concentrations of mouse prolactin [50], progesterone [51] and 17-β-estradiol (E2) [52], followed by NRF2 immunoblotting. Unlike prolactin (Supp. Fig. 2a) and progesterone (Supp. Fig. 2b), E2 significantly increased NRF2 levels (1.5-fold, Fig. 5a), supporting similar studies in breast and prostate cancer cells [[53], [54], [55]]. No effect of E2 was observed on KEAP1, a known NRF2 inhibitor [56] (Supp. Fig. 2c). E2 through binding to its associated receptor ERα, protects β-cells against oxidative stress [52,57,58] and loss of E2 signaling leads to diabetes [[59], [60], [61]]. We then addressed whether E2-induced protective effects in β-cells are NRF2-dependent. For this purpose, we used a pharmacological inhibitor of NRF2, ML385 [62], in a concentration (5 μM) that does not affect cell survival under basal conditions (Supp. Fig. 3a). ML385 inhibited NRF2 signaling as assessed by qPCR of several NRF2 target genes in Min6 β-cells (Supp. Fig. 3b). Min6 β-cells were then incubated with H_2_O_2_, an inducer of oxidative stress, in the presence of E2 and with or without ML385 and cell survival was evaluated using TUNEL assay (Fig. 5b). As expected, and in line with previous studies [52], E2 significantly decreased H_2_O_2_-mediated cell death (69 % compared to DMSO + H_2_O_2_ control). On the other hand, ML385 not only increased H_2_O_2_-mediated cell death (1.5-fold compared to DMSO + H_2_O_2_ control) but also significantly blunted E2 protective effect. Similarly, the remarkable decrease in 4HNE induced by E2 in the presence of H_2_O_2_ was eliminated by the NRF2 inhibitor (Fig. 5c, Supp. Fig. 3c), suggesting that E2 exerts its antioxidant and protective effects through activation of the NRF2 signaling.

## 3 Discussion

These studies show for the first time that the adaptive response of β-cells during pregnancy depends on NRF2 function. We found that maternal rodent β-cells display a mild increase in ROS levels towards mid-pregnancy which correlates with increased expression of NRF2 and of some of its antioxidant target genes. This is consistent with published studies reporting that human pregnancy is accompanied by low levels of circulating ROS that are generated due to accelerated fat metabolism and increased rate of oxygen consumption [11,63]. NRF2 levels are highly regulated by multiple factors, of which the most studied is the KEAP1-Cullin3-E3 ubiquitin ligase dependent NRF2 proteasomal degradation. This regulation is inhibited during oxidative stress as a result of ROS interaction with KEAP1 specific cysteine residues, leading to changes in KEAP1 conformation and to its autophagic degradation [16,64,65]. Our data show no changes in KEAP1 levels in islets of pregnant mice. Yet, we cannot exclude KEAP1 inhibition as a mechanism for NRF2 activation during pregnancy, especially due to the apparent increase in ROS levels during gestation. Another regulator of NRF2 levels is the glycogen synthase kinase-3 (GSK3). GSK3 phosphorylation of NRF2 facilitates the recruitment of β-TrCP-Cullin1 which promotes NRF2 proteasomal degradation [64]. This pathway was recently identified in β-cells [66], but has never been studied during pregnancy. Of note, phosphatidylinositol 3-kinase (PI3K), a kinase which signaling contributes to adaptive expansion of maternal β-cells [67], negatively regulates GSK3β [64,66], and women with GDM have high GSK3β levels in adipose and skeletal muscle tissues [68], raising the possibility that maternal β-cell maladaptation might be mediated by GSK3-dependent NRF2 degradation in β-cells. Other factors that may regulate NRF2 during pregnancy include serotonin, as well as the hepatocyte growth factor (HGF) and its receptor, c-Met, due to their antioxidant properties and roles in stimulating β-cell mass expansion during pregnancy [25,[69], [70], [71], [72]]. Testing the effect of different gestational hormones on NRF2 levels in β-cells revealed that the steroid hormone E2 upregulates NRF2. In support of that, incubation of rat insulinoma β-cells, INS1 cells, with Silibinin, an antioxidant polyphenolic compound, increases expression of NRF2 in an ERα-dependent manner [73]. In prostate cancer cells, ERα upregulates NRF2 levels by direct binding to the *Nrf2* promoter [55]. Yet, data from our RNAseq showed no changes in *Nrf2* mRNA during pregnancy, suggesting that at least in this case, this is not the underlying mechanism. It is possible, that as in breast cancer cells, E2 upregulates NRF2 through activation of the PI3K pathway [53,54]. Future studies are needed to explore the mechanisms that regulate NRF2 in β-cells during pregnancy.

Multiple studies have linked GDM with dysregulation of the NRF2 signaling pathway [74,75]. However, while most of these studies focused on the placenta, umbilical cord, and adipose tissue, none has determined the role of NRF2 in promoting functional β-cell mass expansion and normal glucose homeostasis maintenance during pregnancy. Our results show that β-cell NRF2 dysregulation in pregnant mice leads to a GDM-like phenotype and therefore can be used as a model to study GDM. According to our data, only a specific subset of NRF2 antioxidant target genes were significantly upregulated in pregnancy but downregulated in islets from pregnant βNrf2KO mice. All other antioxidant genes that did not follow this expression pattern were either not affected by pregnancy or not controlled by NRF2. While NRF2 is the focus of this study, it is possible that other antioxidant systems play a role in protecting β-cells to mitigate GDM. For example, activating transcription factor 4 (ATF4), Jun proteins, BTB and CNC homology (BACH) proteins, as well as NRF1 (gene name *Nfe2l1*) and NRF3 (gene name *Nfe2l3*), bind antioxidant response elements, thereby activating the transcription of antioxidant genes [76]. Yet, their role in the adaptation of the β-cell during pregnancy has not been studied. Thus, further studies are needed to explore the role of other antioxidant pathways in the expansion of β-cell mass during pregnancy.

In agreement with our previous studies in mice fed a DIO diet [16], NRF2 contributes to the adaptive expansion of β-cell mass during pregnancy by promoting β-cell proliferation and increasing β-cell survival. Since obesity and pregnancy share common metabolic features, including development of insulin resistance and elevated ROS levels [2,11,16,63,64,77], it is not surprising that in both situations, inhibition of NRF2 in β-cells results in dysregulated glucose homeostasis [16]. Importantly, maternal obesity is a major risk factor for GDM in humans [77]. In rodents, DIO diet feeding during gestation results in maternal hyperglycemia [78], while in their male offspring it is associated with reduced β-cell mass, decreased β-cell function, and elevated islet oxidative stress [78,79]. Of note, although β-cell proliferation is the primary driver of maternal rodent β-cell expansion during pregnancy (as it is in obesity), β-cell neogenesis may also contribute to this expansion, as has been shown in humans [24,80,81]. However, whether NRF2 plays a role in β-cell neogenesis during pregnancy is unknown and need to be studied further.

Here, we also show that NRF2 is essential for preserving GSIS in both pregnant and NP mice. These findings are distinct from our previous study, where *Nrf2* deletion in male mice fed a DIO diet did not show any effect on GSIS [16], suggesting sex differences in the role in NRF2 in β-cell function, an aspect that require additional research. The mitochondria are a primary source of ROS production, which are released as a byproduct of incomplete oxygen reduction during oxidative phosphorylation [64]. Moderate ROS levels stimulate GSIS by increasing calcium flux, a key part of the signal transduction process that triggers insulin release from β-cells [64]. Conversely, high ROS levels inhibit GSIS through direct interaction with free thiols located on top of K_ATP_ channels, various glycolytic enzymes and mitochondrial complex IV [64]. We have previously shown that deletion of *Nrf2* directly affects mitochondrial function by reducing membrane potential, mitochondrial ATP synthesis, and the expression of genes encoding mitochondrial complexes [16,17,82]. Similarly, reduced expression of mitochondrial genes is observed in the current study suggesting that NRF2 supports GSIS during pregnancy by maintaining mitochondrial function. It should be noted that inhibition of NRF2 during pregnancy reduced expression of several calcium and cAMP signaling related genes that play a role in GSIS, including adenylate cyclase 1 (*Adcy1*) [83], type 2 ryanodine receptor (*RyR2*) [84], calcium voltage-gated channel subunit alpha1 E (*Cacna1e*) [85] and calcium voltage-gated channel auxiliary subunit gamma 1 (*Cacng1*) [86]. Thus, we speculate that GSIS is suppressed in islets from NP and GD15 βNrf2KO mice due to dysfunctional mitochondria and altered calcium and cAMP signaling. Future studies are needed to further explore the mechanism by which NRF2 affects these signaling pathways.

Importantly, our work provides the first evidence for an additional mechanism by which NRF2 maintains functional β-cell mass, i.e., by controlling expression of genes that suppress lipid peroxidation, prevent lipid peroxidation-induced ferroptosis and detoxify reactive aldehydes such as 4HNE in β-cells. Free 4HNE molecules that evade detoxification can attract electron-rich amino or thiol groups-containing proteins, perform nucleophilic attack and form irreversible bonds called “protein carbonylation” [38,39,41]. Once a protein is carbonylated, it can undergo misfolding, aggregation, and degradation, leading to its loss of function [[39], [40], [41]]. Not surprisingly, high levels of 4HNE-protein adduct are found in blood and various tissues of T2D patients, including liver, adipose and skeletal muscle, as well as in islets of T2D cadaveric donors [[87], [88], [89]]. Likewise, 4HNE and other lipid peroxidation reactive byproducts are found in serum and plasma of patients with GDM [90]. Studies are needed to examine the role of NRF2 in mitigating β-cell protein carbonylation in GDM.

Overall, the results of this study suggest that NRF2 can serve as a potential therapeutic target for treating GDM. Indeed, the NRF2 pharmacological activator, tertiary butylhydroquinone (THBQ) improves glucose homeostasis in a mouse model of GDM [74,75]. However, whether this is the result of specific effects of this activator in β-cells has not been studied. Notably, prevention of GDM using broad spectrum antioxidants is still controversial. While treatment with N-Acetyl-l-cysteine (NAC), vitamin E and vitamin C have been reported to ameliorate diabetes in GDM and T2D mouse models [[91], [92], [93], [94], [95]], clinical trials in women with GDM are still lacking and the administration of NAC to T2D patients did not improve their glycemia [96]. It might be that the administration of NAC blocked ROS-dependent activation of endogenous NRF2 signaling. Additionally, administration of broad-spectrum antioxidants may eliminate even low levels of ROS, which are beneficial to β-cells at small physiological doses [64]. Whether NRF2 pharmacological activators can induce β-cell mass expansion and regulate glucose homeostasis in pre-clinical mouse models of GDM warrant further studies.

## 4 Methods

### 4.1 Mouse models

Female β-cell specific NRF2 deletion mice were generated by crossing MIP-CreER^TAM^ mice [97] (RRID: IMSR_JAX:024709) with Nrf2^lox/lox^ mice [98] (RRID: IMSR_JAX:025433) as previously described [16]. Female mice were injected intraperitoneally for 5 consecutive days with 75 mg/kg tamoxifen (Tam) (Sigma-Aldrich T5648) dissolved in corn oil. Followed Tam injections, females were let recover for 30 days to allow Tam washout before mating. All studies were performed with the approval of and in accordance with guidelines established by the institutional animal care and use committee of the Icahn School of Medicine at Mount Sinai.

### 4.2 Islet isolation

Mouse islets were isolated after collagenase P (Sigma-Aldrich 11213865001) injection through the pancreatic duct, followed by digestion and separation by density gradient using Histopaque-1077 (Sigma-Aldrich 10771), as previously reported [16].

### 4.3 Immunolabeling

Paraffin-embedded pancreatic sections were immunolabeled for insulin (Genetex GTX39371 or R&D MAB-1417) and Ki67 (Invitrogen MA5-14520) to assess β-cell proliferation; or NRF2 (Cayman Chemicals 10214) to assess β-cell NRF2 levels; or DNA/RNA oxidative damage (Abcam ab62623) to assess β-cell oxidative stress; or 4HNE (Abcam ab48506) to assess β-cell 4HNE levels. The percentage of Ki67/NRF2/oxidative stress/4HNE-positive/insulin-positive events was blindly calculated and quantified from at least 1000 insulin-positive cells per mouse, across at least four islets of varying sizes. For β-cell mass an average of three insulin-stained mouse pancreatic sections, separated by at least 5 μm, were blindly measured using ImageJ software (National Institutes of Health). A minimum of three mice were analyzed per condition.

### 4.4 TUNEL assay

TUNEL labeling was performed according to the manufacturer instructions, using the DeadEnd Fluorometric TUNEL System (G3250; Promega). Samples were then immunostained with insulin antibody (Genetex GTX39371 or R&D MAB-1417). The percentage of TUNEL-positive/insulin-positive events was blindly calculated and quantified from at least 1000 insulin-positive cells per mouse, across at least four islets of varying sizes. A minimum of three mice were analyzed per condition.

### 4.5 Immunoblotting

Mouse islets were digested, protein was extracted and separated into 10 % SDS-PAGE as previously described [16]. Membranes were incubated with NRF2 (Proteintech 16396-1-AP) or KEAP1 (Abcam ab227828) antibodies. Protein levels were quantiﬁed using Image J densitometry and normalized to actin levels.

### 4.6 RNA isolation and RNAseq

Samples were extracted and sequenced, as previously described [17]. Briefly, RNA was extracted from NP/GD15 control/βNrf2KO mouse islets, three/four mice per group by RNeasy Micro Kit (QIAGEN, Germantown, MD, USA). RNA was quantified by Qubit 2.0 Fluorometer (ThermoFisher Scientific, Waltham, MA, USA) and integrity checked with TapeStation 4200 (Agilent Technologies, Palo Alto, CA, USA). rRNA depletion libraries were prepared using QIAGEN FastSelect rRNA HMR Kit (Qiagen, Hilden, Germany) and NEBNext Ultra II RNA Library Preparation Kit for Illumina HiSeq (NEB, Ipswich, MA, USA). Libraries were validated using the Agilent TapeStation 4200 and quantified by Qubit 2.0 Fluorometer and quantitative PCR (KAPA Biosystems, Wilmington, MA, USA). Samples were sequenced by Illumina HiSeq 3000/4000 with a 2x150bp paired-end configuration. Read quality was determined by using fastqc (version 0.11.9). All samples passed the quality assessment and were processed for read alignment to ENSEMBL Mus musculus GRcm39/mm39 by using star aligner (version 2.7.10a) and samtools (version 1.17) for creating the bam files. Raw gene counts were finally generated by using subread (version 2.0.1). Differential gene expression was conducted using edgeR (version 3.40.2) and Limma (version 3.5.4.2) r packages Differentially expressed genes were called at FDR<0.05.

### 4.7 qPCR

Total RNA was extracted as detailed above from islets isolated from mice and qPCR was performed as previously described [16]. Primers sequences are listed in Supp Table 2.

### 4.8 Glucose homeostasis

Blood glucose was determined by glucometer and plasma insulin by ELISA (Mercodia 10-1249-01). For intraperitoneal glucose tolerance tests (IPGTT), mice were fasted for 16 h and then intraperitoneally injected with glucose at a dose of 2 g/kg d-glucose. GSIS and insulin content measurements were performed as previously described [16].

### 4.9 Min6 cell culture and hormones stimulation

Min6 β-cells were grown in DMEM (Gibco 11965–092) media supplemented with 1 % P/S (Gibco 15140–122) and 15 % Fetal bovine serum (Corning 35-011-CV). For E2 experiments, growth media was replaced with glucose-free DMEM (Gibco 11966–025) supplemented with 5.5 mM glucose, 1 % P/S and 15 % Fetal bovine serum for 6 h. Cells were then washed twice with PBS and incubated with Phenol red-free glucose-free DMEM (Gibco A14430-01) supplemented with 5.5 mM glucose, 15 % charcoal-stripped serum (Gibco A33821-01), 1 % P/S and E2 (Sigma-Aldrich E2758) at various concentrations for 18 h (for immunoblotting) or 72 h (for TUNEL and 4HNE immunolabeling) as previously described [52]. Similar conditions were used for progesterone (Sigma-Aldrich P8783) experiments. For prolactin experiments, cells washed twice with PBS and incubated with serum-free DMEM supplemented with 5.5 mM glucose and 1 % P/S for 16 h. Media was then replaced to a similar media supplemented with 1 % Bovine serum albumin (BSA) and Mouse prolactin (Sigma-Aldrich SRP4688, diluted in serum-free DMEM media containing 0.1 % BSA) for 6 h, as previously described [50,99,100].

For survival assays and 4HNE immunolabeling, cells were added with a pharmacological inhibitor of NRF2, ML385 [62] (Sigma-Aldrich SML1833) for 72 h or 100 μM H_2_O_2_ for 6 h.

### 4.10 Statistical analysis

Data are presented as means ± SEM. The number of biologically independent replicates (n) for each experiment are indicated in the figure legends. Statistical analysis was performed using unpaired two-tailed *t*-test, one-way ANOVA, and two-way ANOVA with Tukey multiple comparison test using GraphPad Prism (version 8.4.3). A p value < 0.05 was considered statistically significant.

## CRediT authorship contribution statement

**Fatema Haidery:** Visualization, Investigation, Formal analysis. **Luca Lambertini:** Writing – review & editing, Software, Investigation, Formal analysis. **Isabelle Tse:** Investigation, Formal analysis. **Sriya Dodda:** Investigation, Formal analysis. **Adolfo Garcia-Ocaña:** Writing – review & editing, Funding acquisition, Conceptualization. **Donald K. Scott:** Writing – review & editing, Funding acquisition, Conceptualization. **Sharon Baumel-Alterzon:** Writing – original draft, Validation, Supervision, Methodology, Investigation, Funding acquisition, Formal analysis, Conceptualization.

## Authors’ relationships and activities

The authors declare that there are no relationships or activities that might bias, or be perceived to bias, their work.

## Funding

This study was supported by the 10.13039/100000002National Institutes of Health, 10.13039/100000062National Institute of Diabetes and Digestive and Kidney Diseases, Mentored Research Scientist Development Award K01 DK128387-01 (to SBA) and R01DK139631 (DKS and 10.13039/100021277AGO).

## Declaration of competing interest

None.

## Data Availability

Data will be made available on request.

## Appendix A Supplementary data

Supp. Fig. 1: (a) Expression levels (in logCPM values) of *Nrf2* in islets from NP or GD15 female mice. (b) Islets from NP or GD15 female mice were subjected to immunoblotting using a KEAP1 and actin antibodies. KEAP1 levels were quantified using ImageJ software. (c) The number of embryos of βNrf2KO and control mice were counted. (d) Expression heatmap of logCPM values for selected cell cycle or (e) mitochondrial genes. Data are means ± SEM for n = 5–11.

Supp. Fig. 2: (a) Min6 β-cells were incubated with DMSO or mouse prolactin for 6 h or (b) progesterone for 18 h, and immunoblotting using NRF2 antibody was performed. (c) Min6 β-cells were incubated with DMSO or E2 for 18 h and immunoblotting using KEAP1 antibody was performed. NRF2 and KEAP1 levels were quantified using ImageJ software. Data are means ± SEM for n = 3–4.

Supp. Fig. 3: (a) Min6 β-cells were incubated with DMSO or ML385 for 72 h and the number of viable cells was counted. (b) Min6 β-cells were incubated with DMSO or 5 μM ML385 for 72 h and high glucose (25 mM) was added at the last 6 h. The expression level of NRF2 target genes was assed using qPCR. (c) Min6 β-cells were incubated with DMSO or 5 μM ML385, in normal (5 mM) or high (25 mM) glucose for 16 h. As a positive control, cells were incubated with 100 μM H2O2 for 6 h. Immunolabeled for 4HNE and insulin (scale bar 200 μM) was performed. Data are means ± SEM for n = 3–5. ∗∗*p* < 0.01, ∗∗∗*p* < 0.001.
