# Supplementary figures and images for: NRF2 deficiency leads to inadequate beta cell adaptation during pregnancy and gestational diabetes
Source: Redox Biol. 2025 Feb 24;81:103566. doi: 10.1016/j.redox.2025.103566 (PMC11930207; doi:10.1016/j.redox.2025.103566)

## Slide 1
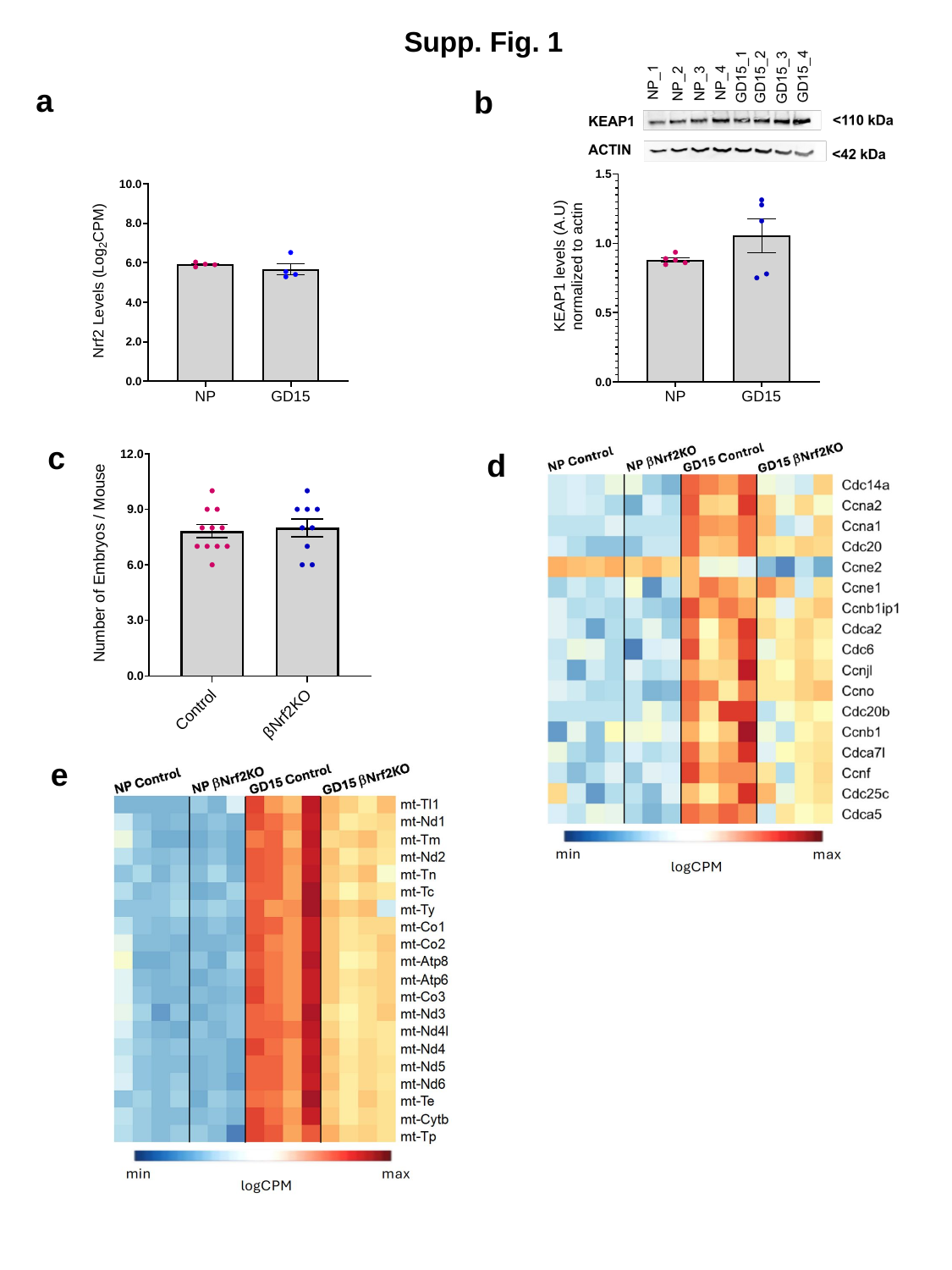

Supp. Fig. 1
a
b
c
d
e

## Slide 2
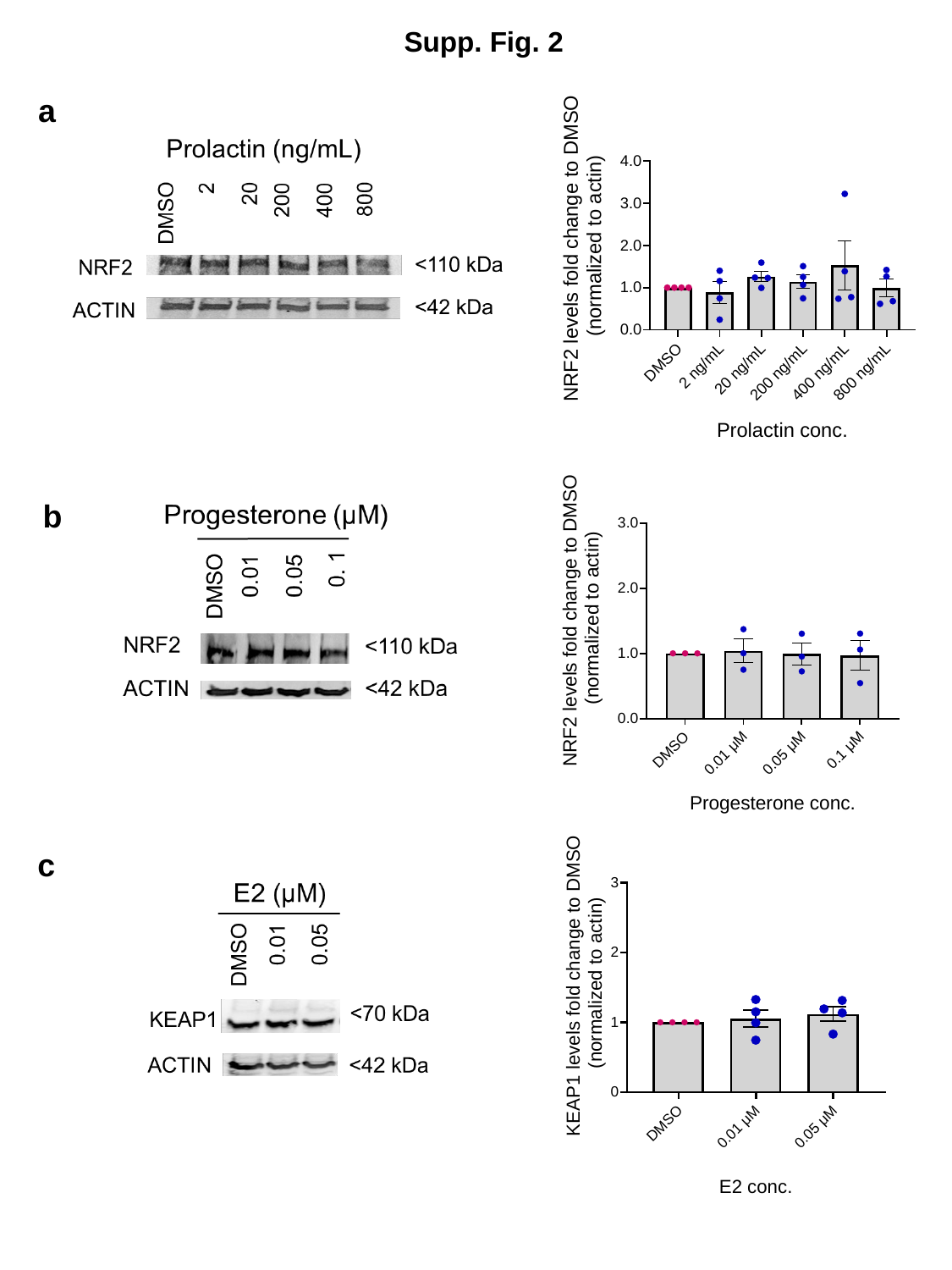

Supp. Fig. 2
a
b
c

## Slide 3
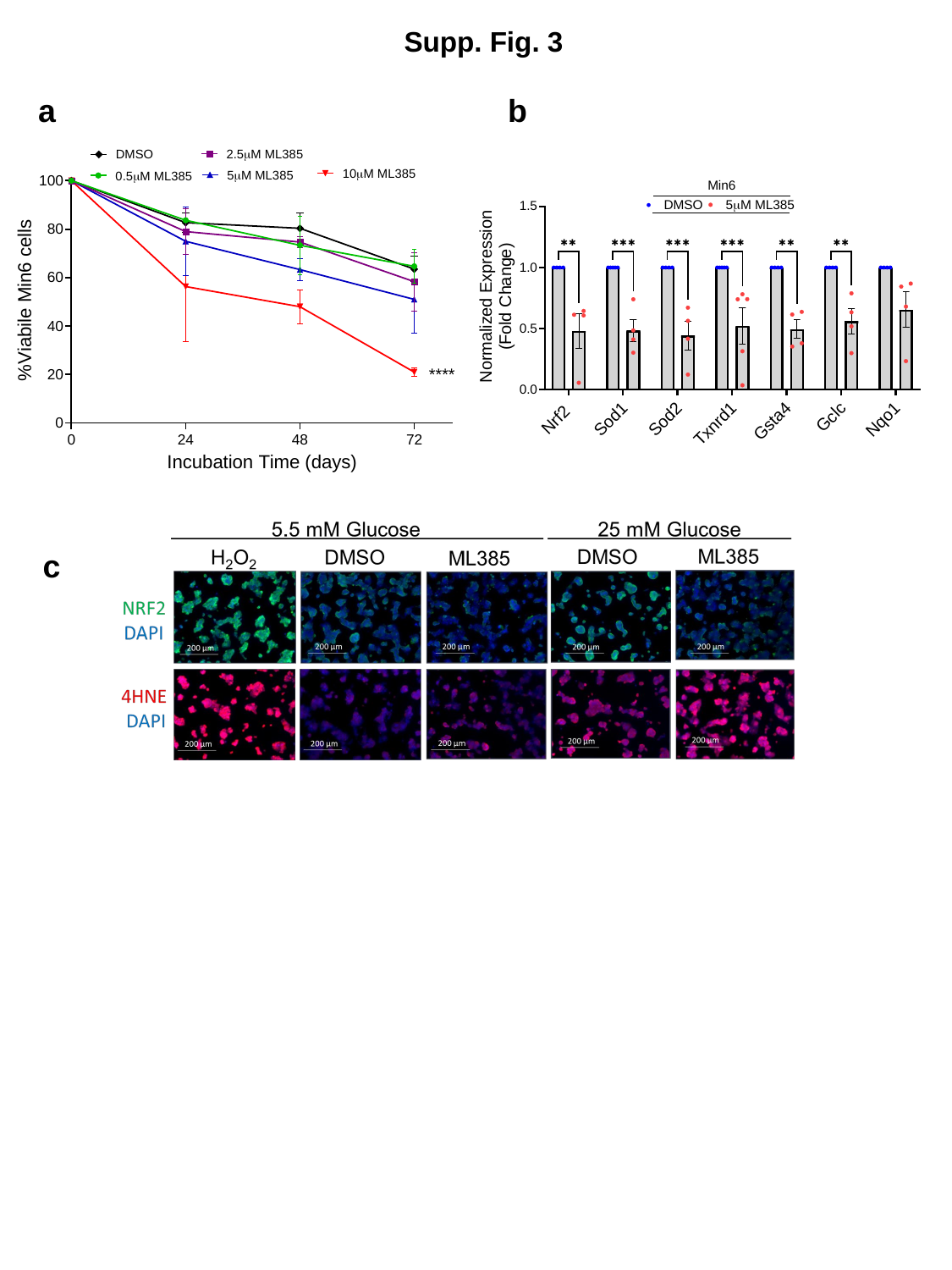

Supp. Fig. 3
a
b
c

Supplement: Multimedia component 1 [file mmc1.pptx]
