# Supplementary material for: NRF2 deficiency leads to inadequate beta cell adaptation during pregnancy and gestational diabetes
Source: Redox Biol. 2025 Feb 24;81:103566. doi: 10.1016/j.redox.2025.103566 (PMC11930207; doi:10.1016/j.redox.2025.103566)
Supplement: Multimedia component 3 [file mmc3.docx]

**Supp. Table 2: List of primers used in this paper for qPCR**

| **Gene Name** | **Forward sequence** | **Reverse Sequence** |
| --- | --- | --- |
| Actin | AGCCATGTACGTAGCCATCC | CTCTCAGCTGTGGTGGTGAA |
| Nrf2 | AGGACATGGAGCAAGTTTGG | TTCTTTTTCCAGCGAGGAGA |
| Sod1 | AACCAGTTGTGTTGTCAGGAC | CCACCATGTTTCTTAGAGTGAGG |
| Sod2 | TGGACAAACCTGAGCCCTAAG | CCCAAAGTCACGCTTGATAGC |
| Txnrd1 | GTGGCGACTTGGCTAATC | ACCAGGAGAGACACTCAC |
| Gsta4 | GGGAACAGTATGAGAAGATGCAAAA | CCCATCGATTTCAACCAAGG |
| Gclc | GGACAAACCCCAACCATCC | GTTGAACTCAGACATCGTTCCT |
| Nqo1 | GAAGGAGGCTGCTGTAGAGG | ATCACCAGGTCTGCAGCTTC |
